# Short-term restoration practices change the bacterial community in degraded soil from the Brazilian semiarid

**DOI:** 10.1038/s41598-024-57690-y

**Published:** 2024-03-21

**Authors:** Davila Esmelinda Oliveira Silva, Romario Martins Costa, Janaira Rocha Campos, Sandra Mara Barbosa Rocha, Arthur Prudencio de Araujo Pereira, Vania Maria Maciel Melo, Francisca Andrea Silva Oliveira, Francisco de Alcantara Neto, Lucas William Mendes, Ademir Sergio Ferreira Araujo

**Affiliations:** 1 Soil Microbial Ecology Group, Federal University of Piauí, Teresina, PI Brazil; 2 Federal University of Ceara, Fortaleza, CE Brazil; 3 Plant Science Department, Federal University of Piauí, Teresina, PI Brazil; 4 Center for Nuclear Energy in Agriculture, University of Sao Paulo, Piracicaba, SP Brazil; 5 Soil Quality Lab., Agricultural Science Center, Federal University of Piauí, Teresina, PI Brazil

**Keywords:** Land degradation, Amplicon sequencing, Semiarid soils, Soil microbial ecology, Environmental sciences, Microbial ecology

## Abstract

Land degradation by deforestation adversely impacts soil properties, and long-term restoration practices have been reported to potentially reverse these effects, particularly on soil microorganisms. However, there is limited knowledge regarding the short-term effects of restoration on the soil bacterial community in semiarid areas. This study evaluates the bacterial community in soils experiencing degradation (due to slash-and-burn deforestation) and restoration (utilizing stone cordons and revegetation), in comparison to a native soil in the Brazilian semiarid region. Three areas were selected: (a) under degradation; (b) undergoing short-term restoration; and (c) a native area, and the bacterial community was assessed using 16S rRNA sequencing on soil samples collected during both dry and rainy seasons. The dry and rainy seasons exhibited distinct bacterial patterns, and native sites differed from degraded and restoration sites. Chloroflexi and Proteobacteria phyla exhibited higher prevalence in degraded and restoration sites, respectively, while Acidobacteria and Actinobacteria were more abundant in sites undergoing restoration compared to degraded sites. Microbial connections varied across sites and seasons, with an increase in nodes observed in the native site during the dry season, more edges and positive connections in the restoration site, and a higher occurrence of negative connections in the degradation site during the rainy season. Niche occupancy analysis revealed that degradation favored specialists over generalists, whereas restoration exhibited a higher prevalence of generalists compared to native sites. Specifically, degraded sites showed a higher abundance of specialists in contrast to restoration sites. This study reveals that land degradation impacts the soil bacterial community, leading to differences between native and degraded sites. Restoring the soil over a short period alters the status of the bacterial community in degraded soil, fostering an increase in generalist microbes that contribute to enhanced soil stability.

## Introduction

Land degradation poses a significant contemporary threat, with a substantial increase observed worldwide. According to the United Nations, approximately 25% of the world’s land is undergoing a degradation process, impacting around 3.2 billion people^1^. This process not only affects agriculture and the environment but also contributes to social and economic instability^2^. Specifically, it has led to substantial losses in biodiversity^3^, particularly within the soil ecosystem^4^. On the other hand, restoration efforts are challenging and require a considerable amount of time^5^. Despite these challenges, land restoration has the potential to enhance soil and plant productivity, sequester carbon, and restore soil biodiversity^6^. Previous studies have observed a long-term positive effect of restoration on degraded lands worldwide, emphasizing its crucial role in promoting sustainable ecosystems^7,8^.

One of the most abundant and diverse components of soil biodiversity is bacteria, which plays essential roles in soil functioning^9^. However, they are significantly and negatively impacted by land degradation^10^. On the other hand, the restoration process can have positive effects on the soil bacterial community^7,11,12^. For example, Ma et al.^11^ observed increased bacterial diversity and altered community compositions, similar to those found in native ecosystems, in an area undergoing land restoration in China. Hence, a better understanding of the impact of restoring degraded areas on microbial communities is crucial for a more sustainable use of the soil^13^.

Brazil has a semiarid region undergoing significant degradation. Specifically, areas in the Brazilian Northeast, known as the Nucleus of Degradation of Gilbues (Piauí state) and Irauçuba (Ceara state), face environmental challenges. The causes of degradation in Gilbues include both deforestation and mining, while Irauçuba experiences degradation due to overgrazing^13^. Despite these challenges, these areas have been subjected to different restoration strategies since the 2000s, such as green manuring and grazing-exclusion, respectively. These strategies have shown promising results in terms of the recovery of soil microbial communities^7,14,15^. However, these degraded areas in the Brazilian semiarid region have undergone long-term restoration, spanning two decades. Yet, the responses of soil microbial communities in areas undergoing short-term restoration, such as those belonging to the Recovery Units of Degraded Areas and Reduction of Climate Vulnerability (URAD) initiative, remain unknown.

**Figure 1:**
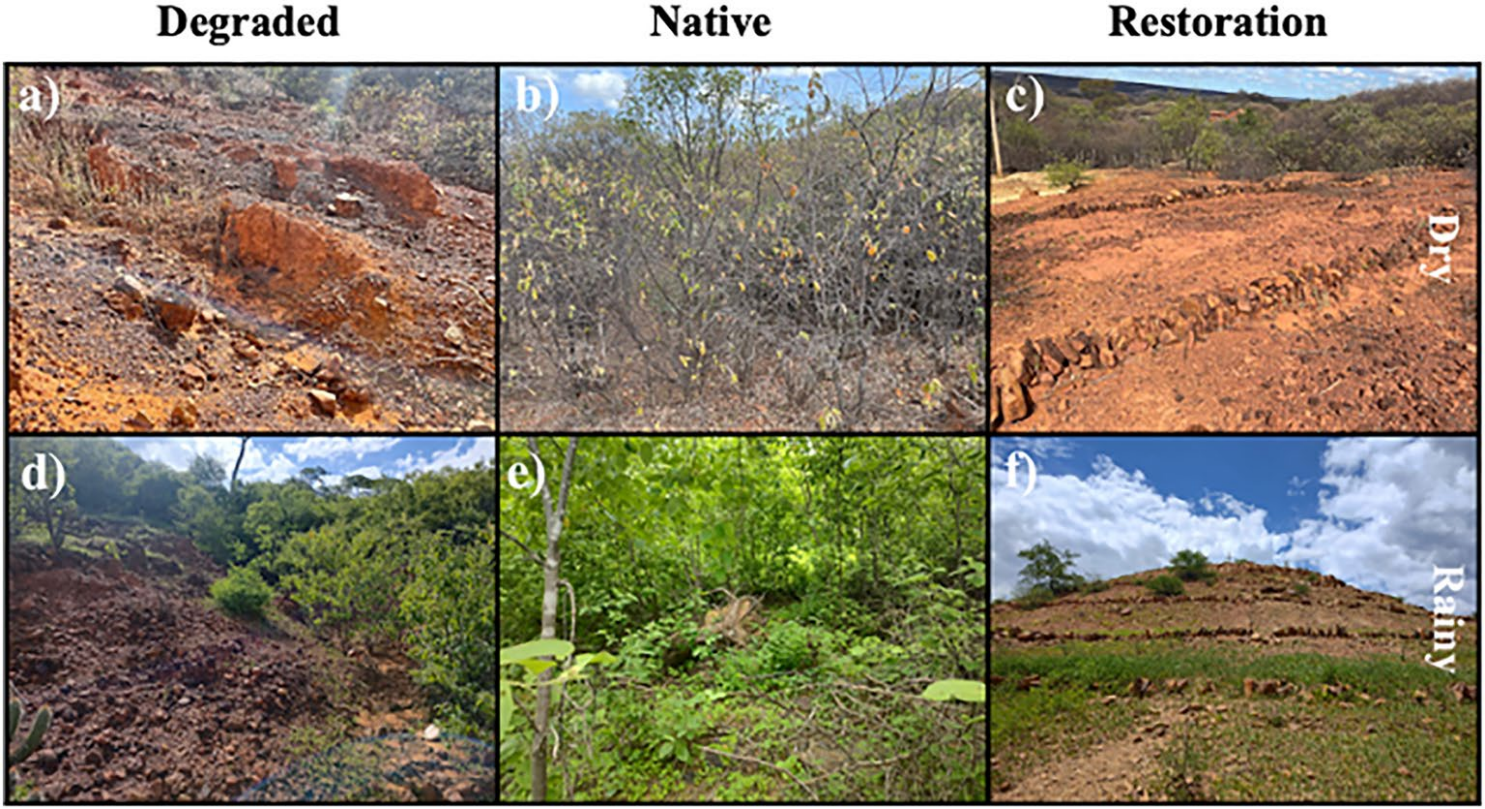
Landscape scenarios: Degraded (**a** and **d**), native (**b** and **e**), and under restoration showing the stone cords (**c** and **f**) areas during dry and rainy seasons.

The implementation of URAD has received support from the Brazilian government, which has invested approximately US$10 million in applying strategies to restore this degraded land^16^. The primary environmental strategy involves the construction of stone cords (Fig. 1) for water and soil conservation, aimed at preventing soil erosion and maintaining soil moisture. This approach contributes to the recovery of soil biological diversity and creates conditions for food production. With controlled soil erosion and maintained moisture, revegetation practices are employed, utilizing native plant species such as *Anadenanthera colubrina*, *Amburana cearensis*, *Cnidoscolus phyllacanthus*, and *Mimosa caesalpiniaefolia*. These species are associated with local crops, such as maize and beans, to restore vegetation cover. Therefore, during the last two years of implementation in the Brazilian semiarid region, URAD has contributed to both social and economic improvements for the local population^16^. However, from an environmental perspective, no assessment has been conducted thus far, including an evaluation of soil biodiversity. Since the strategies implemented in URAD have been applied in the short term (since 2020), it remains unclear whether these practices have had an impact on changing the soil bacterial community compared to degraded soil in the Brazilian semiarid region. According to Pereira et al.^7^, the bacterial community is sensitive and can reflect the effects of both degradation and restoration, especially in arid and semiarid conditions.

To enhance our understanding regarding the impact of restoring degraded areas on microbial communities, this study hypothesized that (1) land degradation changes the soil bacterial community, and (2) restoration efforts applied in URAD could, in the short term, be effective in restoring that bacterial community to a similar status as the native area. Thus, this study aimed to assess the structure, diversity, and composition of the soil bacterial community in soil undergoing degradation (deforestation by slash-and-burn) and restoration (construction of stone cords and revegetation practices), in comparison to a native environment in the Brazilian semiarid region.

## Results

**Figure 2:**
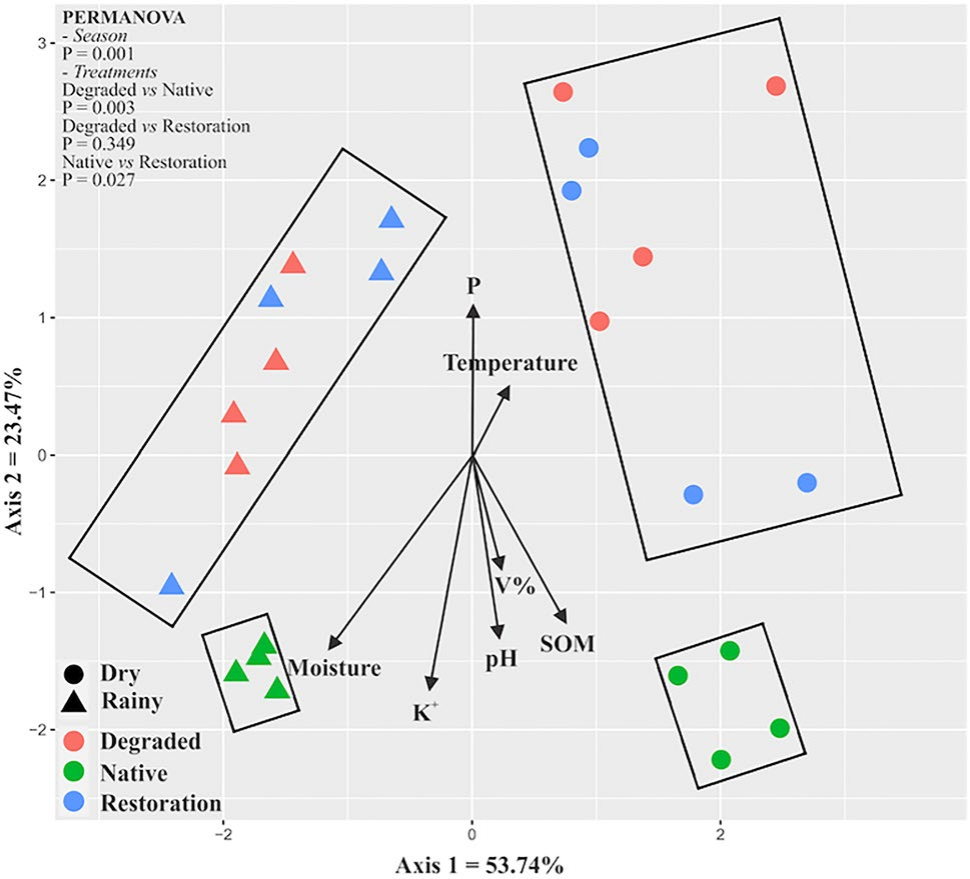
Non-metric multidimensional scaling (NMDS) comparing microbial groups in degraded, native, and under restoration areas during dry and rainy seasons. PERMANOVA test was adopted to test the significance of groups clusterization among treatments and seasons.

The structure of the soil bacterial community changed when comparing sites and seasons. A clear and significant separation between the dry and rainy seasons was observed in the bacterial community’s structure. Within each season, there was a distinct separation between native sites compared to both sites under degradation and restoration, which clustered together. Redundancy analysis revealed that certain soil parameters drove the responses of the bacterial community (Fig. 2). In general, phosphorus content and temperature influenced the bacterial community in both degraded and restoration sites. In contrast, soil moisture, soil organic matter (SOM), pH, potassium content, and vanadium percentage influenced the bacterial community in the native site.

**Figure 3:**
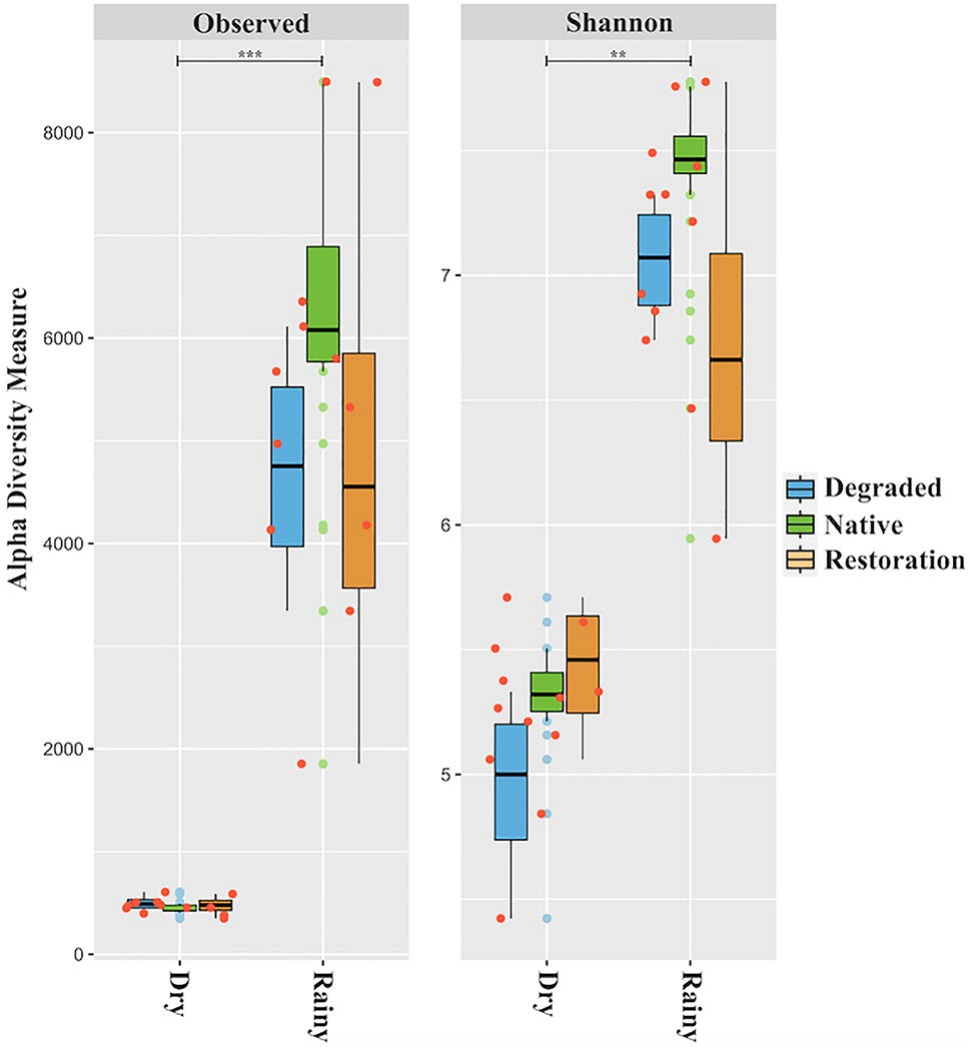
Alpha diversity measurement (i.e., observed ASVs, and Shannon indexes) in degraded, native, and under restoration areas during dry and rainy seasons. The Kruskal–Wallis test (*p* < 0.05*, 0.01*, 0.001**) was used to determine whether there are statistically significant differences among seasons and treatments.

The diversity indices were also influenced by sites and seasons (Fig. 3). Regardless of the site, the lowest values for observed ASVs and Shannon indices were found during the dry season. During this period, no significant differences were observed among all sites for the observed ASVs, while the Shannon index showed higher values in both sites under native forest and restoration. In the rainy season, the site under native forest presented the highest values for observed ASVs and Shannon indices, while both sites under degradation and restoration did not differ significantly.

**Figure 4:**
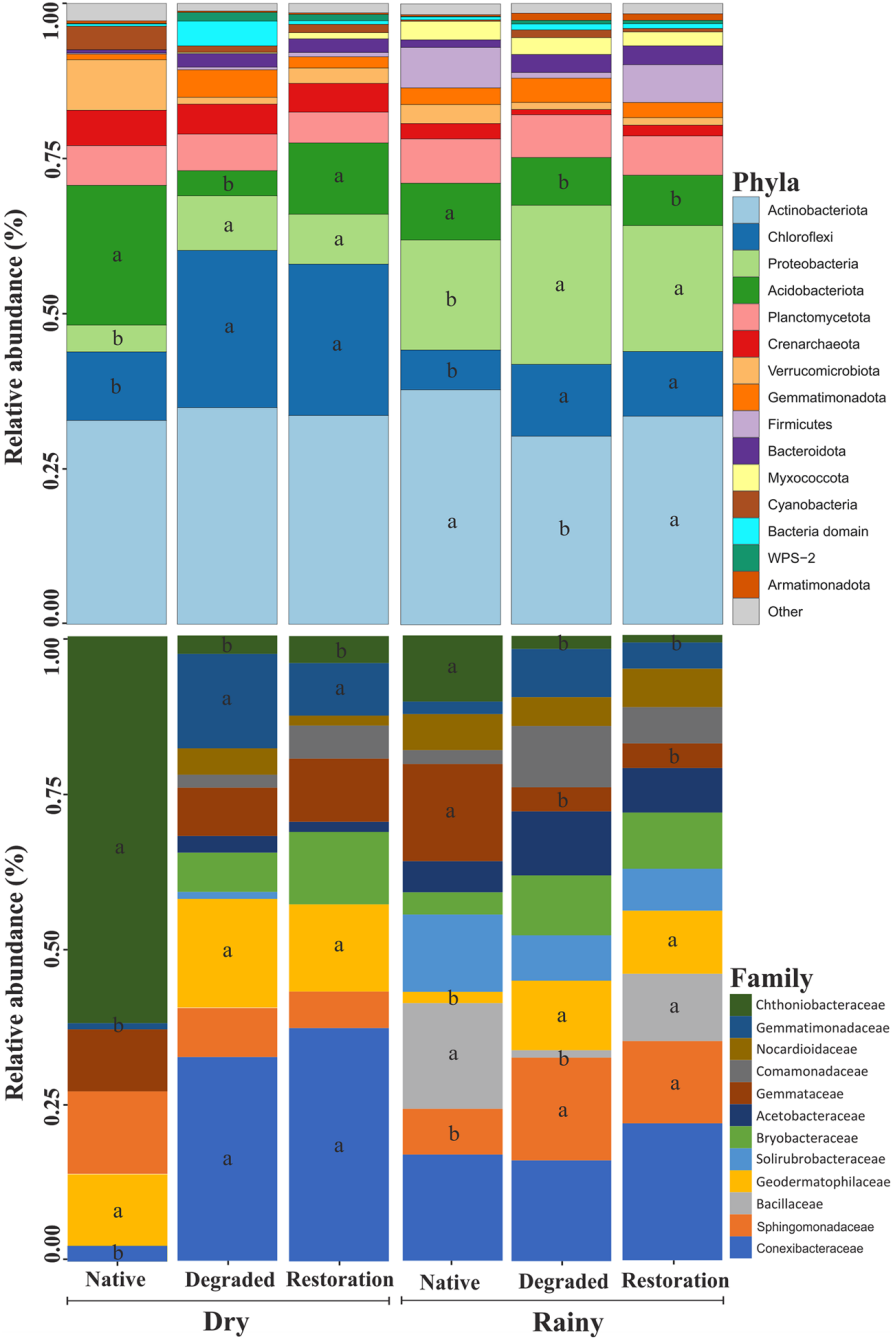
Microbial composition (relative abundance) of the main phyla and family in degraded, native, and under restoration areas during dry and rainy seasons. The Kruskal–Wallis test was used to determine whether there are statistically significant differences among seasons and treatments. Lower cases compared treatments inside each season.

In general, Actinobacteria Chloroflexi, Proteobacteria, Acidobacteria, and Planctomycetota were the most abundant phyla observed in all sites (Fig. 4). Interestingly, the relative abundance of the main phyla changed according to evaluated sites. For instance, in both seasons, the sites under degradation and restoration showed a higher abundance of Chloroflexi and Proteobacteria than the native site (*P* < 0.05). In contrast, the native site showed a higher abundance of Acidobacteria as compared to others (*P* < 0.05). In the dry and rainy seasons, the site under restoration exhibited a higher abundance of Acidobacteria and Actinobacteria, respectively, compared to degraded sites (*P* < 0.05). At the family level, areas under degradation and restoration showed a higher abundance of Conexibacteraceae, Geodermatophilaceae, and Gematimonadaceae in the dry season (*P* < 0.05). In the rainy season, the areas under degradation and restoration displayed a higher abundance of Sphingomonadaceae and Geodermatophilaceae. In contrast, the native soil showed a higher abundance of Bryobacteraceae (dry season) and Chthoniobacteraceae (rainy season). When comparing sites under degradation and restoration, we observed a higher abundance of Bacillaceae in the site under restoration, similar to the native area.

**Figure 5:**
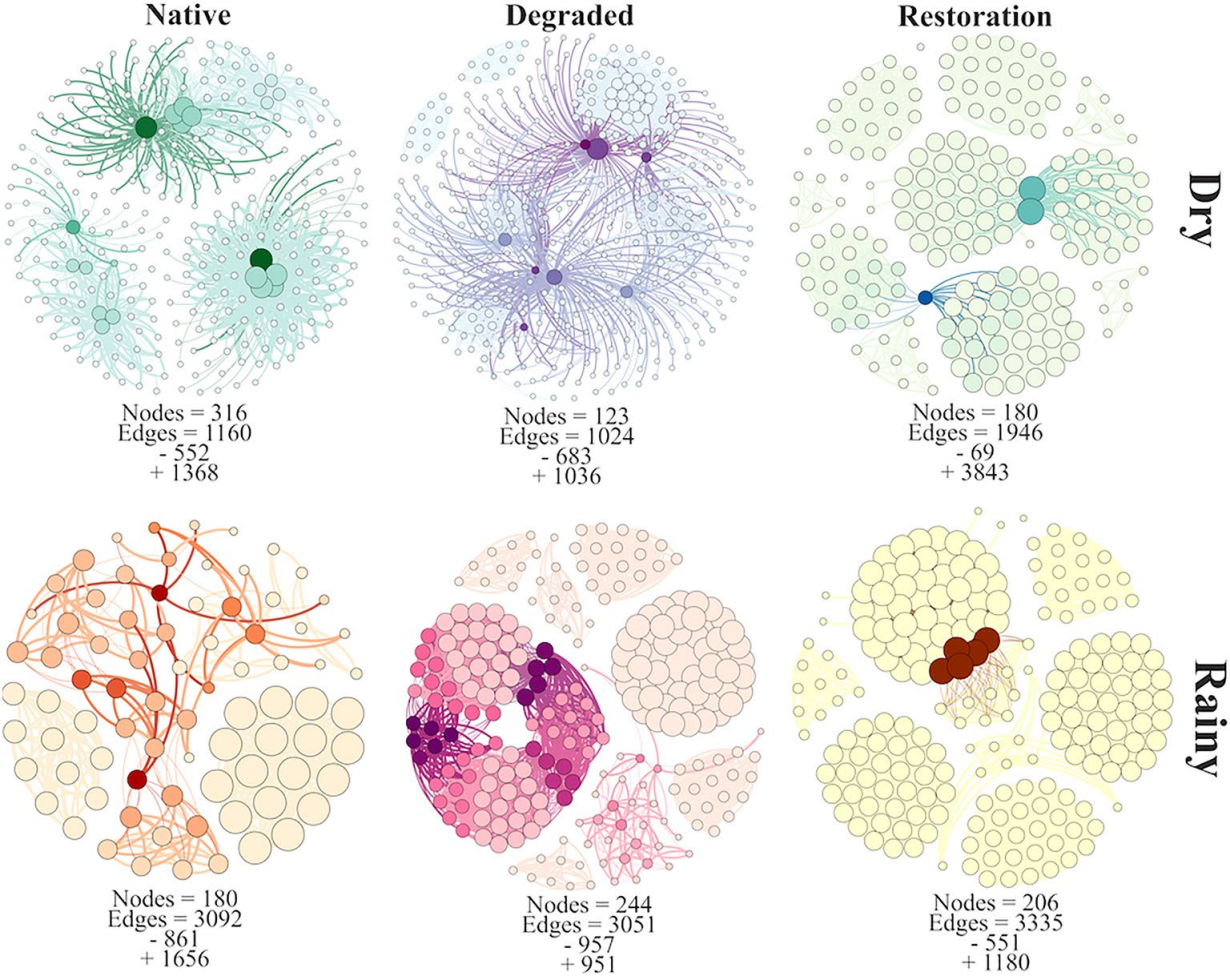
Microbial networks (SparCC methodology) in degraded, native, and under restoration areas during dry and rainy seasons. The − and + mean negative and positive interaction between microorganisms, respectively. The network construction was made using Gephi software.

The co-occurrence network analysis revealed differences when comparing the sites and seasons (Fig. 5). In the dry season, the native vegetation exhibited a higher number of nodes (316 nodes), whereas the site under restoration displayed a higher number of edges (1946 edges) and positive connections (+ 1036). In the rainy season, the site under degradation demonstrated a higher number of nodes (244 nodes) and, notably, displayed the highest number of negative connections (− 683 and − 957 in the dry and rainy seasons, respectively) compared to the other sites.

**Figure 6:**
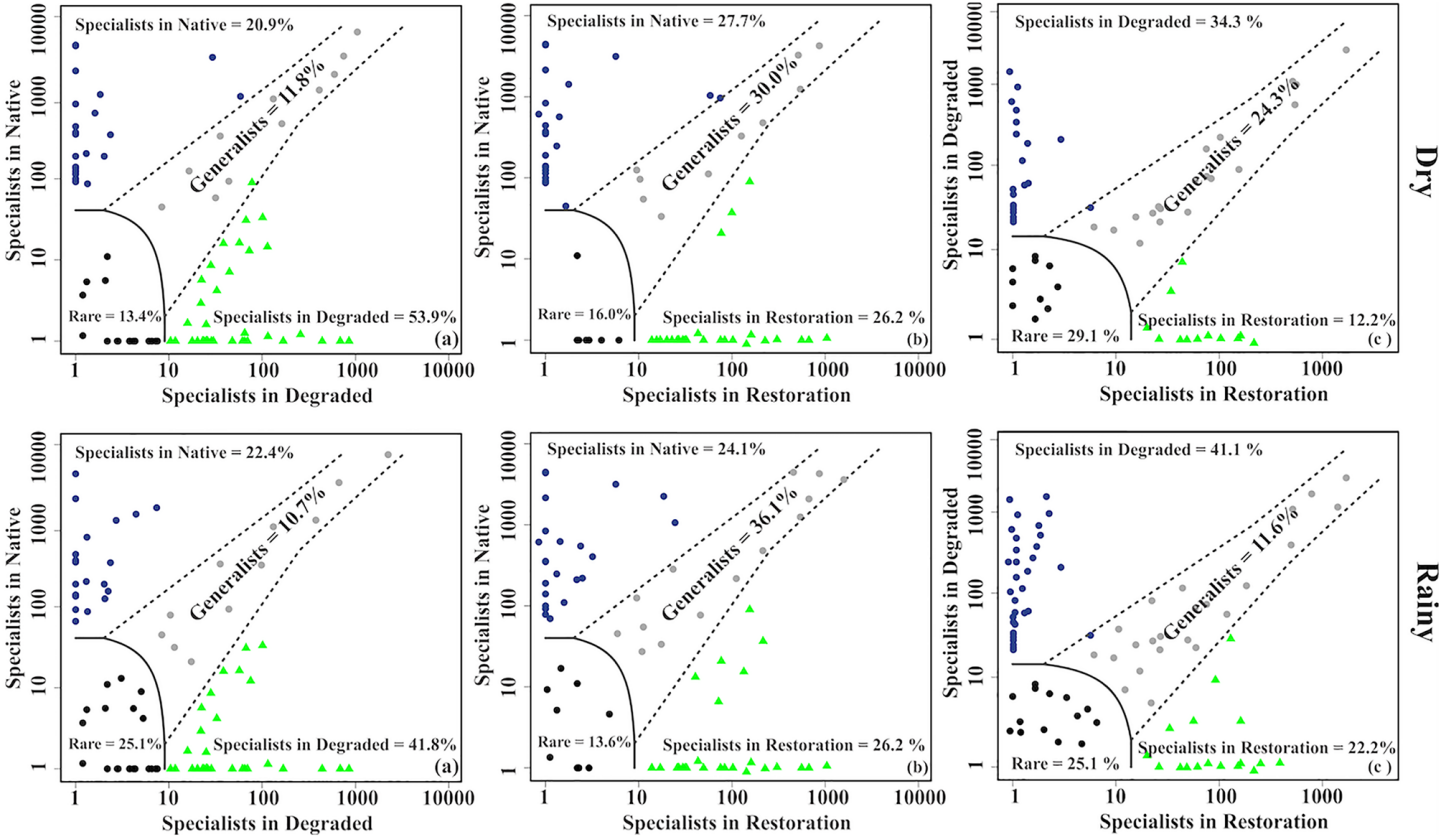
Multinomial species classification method (CLAM) for the niche occupancy test based on pairwise comparison in degraded, native, and under restoration areas during dry and rainy seasons.

Finally, we analyzed the microbial niche occupancy, and the results revealed that degradation decreased the proportion of generalists while increasing the proportion of specialist microbes (Fig. 6). When comparing native and degraded sites, the proportion of generalists was lower (11.8% and 10.7% in the dry and rainy seasons, respectively) than specialists, with a higher proportion of specialists observed in degraded sites (53.9% and 41.8% in the dry and rainy seasons, respectively). Comparing the native site with those under restoration, generalists outnumbered specialists in both dry and rainy seasons. The comparison between sites under degradation and restoration showed a higher proportion of specialists in degraded sites (34.3% and 41.1% in the dry and rainy seasons, respectively); however, this proportion decreased in sites under restoration (12.2% and 22.2% in the dry and rainy seasons, respectively).

## Discussion

In this study, we investigated the bacterial community in soils under native forest, degraded areas, and areas undergoing restoration. The results revealed a distinct community structure among the sites. The anticipated disparity between sites under native forest and the degraded area aligns with expectations, as land degradation typically alters soil conditions and impacts microbial communities^10^, particularly in semiarid regions^7,15^. Similarly, differences were expected between the dry and rainy seasons due to the significant influence of higher soil moisture and lower temperatures in the rainy season, which are more conducive to microbial activity. As suggested by Li et al.^17^ and Lacerda-Junior et al.^18^, soil moisture and temperature emerge as primary factors driving variations in soil microbial communities. On the other hand, we did not observe a clear effect of restoration on altering the bacterial structure compared to degraded soil. This may be attributed to the short-term impact of restoration practices, approximately over 3 years, which may not have been sufficient to induce significant changes in the structure of the soil bacterial community. It is plausible that ecological redundancy, where the decrease or increase of one species can be compensated by others, played a role in maintaining a similar bacterial structure observed in both degraded and restored sites. A prior study indicated that the structure of the soil microbial community might not undergo significant changes in the short term, while other microbial ecological properties may be restored over the years following a land use change^19^. Long-term investigations in restored sites within the Brazilian semiarid have shown substantial changes in the structure of the soil bacterial community two decades after the implementation of restoration practices^7,14,15^. Our results imply that several years may not be adequate to prompt alterations in the structure of the soil bacterial community in response to land degradation.

The bacterial richness and diversity exhibited a significant decrease in degraded sites when compared to native forests. The main process of land degradation observed in our study is through soil erosion, which contributes to losses of soil organic matter and nutrients^20^. These conditions have a negative effect on soil microbial diversity^21^ and alter the structure of the soil bacterial community^22^. Previous studies have consistently reported a negative effect of soil erosion on soil microbial diversity^23,24^. In this sense, the restoration practice implemented in URAD aims to counteract soil erosion and restore soil conditions. However, it is crucial to recognize that significant changes in soil conditions may take time to manifest after the application of the restoration strategy. Indeed, our results indicated that soil bacterial richness and diversity did not exhibit significant differences when comparing sites under land degradation and restoration.

The analysis of the bacterial community composition revealed a higher abundance of Chloroflexi and Proteobacteria phyla in the sites under degradation and restoration, indicating a preference for these phyla in degraded environments. The elevated abundance of Chloroflexi can be attributed to the remarkable adaptability of this phylum to unfavorable conditions, particularly in degraded lands^25^. As for Proteobacteria, this phylum is recognized for its adaptive traits in response to degraded environments^26^.

On the other hand, the restoration practices have not yet been sufficient to alter the dominance of these phyla. A prior study noted that, after four years of restoring degraded land through grazing exclusion, no observable changes were noted in soil microbial composition, and Chloroflexi remained abundant in both degraded and restored lands^27^. Interestingly, our results indicated that both sites under restoration and native vegetation exhibited a higher abundance of Acidobacteria and Actinobacteria, respectively, in both dry and rainy seasons compared to degraded sites. This suggests that restoration efforts can restore the abundance of Acidobacteria and Actinobacteria, similar to the conditions observed in the native area. Acidobacteria is a group of bacteria commonly found in native soil^28^, particularly in tropical regions, and plays a crucial role in various soil functions^29^. Our results imply a negative impact of degradation on the abundance of Acidobacteria, aligning with findings from a previous study assessing land degradation^30^.

In a more detailed analysis at the family level, we observed an increased abundance of Conexibacteraceae and Gematimonadaceae in the dry season and Sphingomonadaceae in the rainy season. Intriguingly, Geodermatophilaceae exhibited a higher abundance in both seasons. This bacterial family possesses the capability to produce biofilm^31^a crucial attribute for thriving in harsh conditions, such as degraded lands. The practice of restoration led to an increased abundance of Bacillaceae compared to the degraded area, suggesting that restoration facilitated the recovery of this bacterial family, similar to native soil. This is noteworthy as Bacillaceae encompasses a bacterial family with genera involved in various mechanisms related to nutrient cycling and the promotion of plant growth^32^.

Next, we evaluated the complexity of community interactions through co-occurrence network analysis, offering insights into the relationships within microbial groups and shedding light on soil microbial ecology^33^. However, it’s essential to acknowledge that the relationships within microbial communities are influenced by soil environmental factors, such as soil moisture and temperature^34^, as well as soil cover and organic matter^7^. These factors could account for the variations observed in the co-occurrence network in both dry and rainy seasons across the sites in our study. The results obtained in the dry season indicated a greater number of nodes in the site under native vegetation, suggesting that this condition preserves more keystone species and their connectivity^24^. Preserving soil conditions in native forests can consequently uphold the interactions, complexity, and stability of soil microbial communities^35^. A more stable and complex ecosystem exhibits a higher capacity to withstand environmental changes^36^. On the other hand, the site under restoration exhibited higher connectivity with positive interactions, indicating that restoration efforts may contribute to restoring the ecological status of soil microbial communities. This heightened microbial connectivity implies the exchange of information between keystone species^35^, a crucial aspect for the soil under restoration to regain functionality, particularly in terms of nutrient cycling^36^. The results observed in the rainy season indicated that the site under degradation had a greater number of nodes; however, these nodes displayed higher negative connections. This outcome implies potential competition between keystone species, which could potentially compromise the functionality of the soil^37^.

Finally, we assessed the proportion of generalist and specialist microbes among the different sites, noting a higher proportion of generalists in soil under native conditions, while specialists were more prevalent in soil under degradation. The primary distinction between generalist and specialist microbes lies in their adaptability to environmental changes, with generalists being more tolerant than specialists. This adaptability allows generalists to maintain community stability when exposed to environmental fluctuations^38^. In degraded lands, there was a reduction in generalist microbes coupled with an increase in specialist microbes, driven by specific conditions induced by soil degradation. This observation could elucidate the elevated proportion of specialist microbes in sites under degradation. Consequently, these results suggest a negative impact of degradation on generalist microbes, leading to a homogenization of the microbial community^39^ and a diminished versatility of microorganisms^40^. On the other hand, restoration efforts have played a role in diminishing the high proportion of specialists found in degraded lands, concurrently increasing the number of generalists. Therefore, over the long term, restoration practices could contribute to augmenting the presence of generalists, thereby aiding in the recovery of soil ecological stability.

## Conclusions

This study demonstrates that land degradation significantly alters the soil bacterial community, showcasing distinct differences when compared to a native site. Consequently, soils under degradation exhibit lower microbial interactions and more negative connections, implying microbial competition and reduced functionality. Furthermore, the restoration efforts hold the potential to recover the abundance of Acidobacteria and Actinobacteria, while concurrently increasing the proportion of generalist microbes, which play a pivotal role in enhancing soil ecological stability. The findings emphasize that restoration practices, such as implementing stone cords to mitigate soil erosion, maintaining soil organic matter and moisture, and incorporating revegetation, influence crucial factors like soil temperature, moisture, and organic matter—key drivers of soil bacterial community dynamics. Moreover, the use of stone cords as a restoration technique can be extrapolated to address potential soil degradation by erosion in areas characterized by high slopes and erodibility. Future studies should delve into the long-term effects of restoration on soil microbial communities and their functional roles. Continuous monitoring of soil properties and microbial communities is deemed essential for effective land management strategies aimed at restoring degraded lands in semiarid regions.

## Methods

### Unit of recovery of degraded areas and reduction of climate vulnerability (URAD)

In this study, we assessed sites belonging to URAD (Fig. 1), located at Santo Antonio Lisboa, a semiarid region from Piauí state, Brazil (6° 58′ 51′′ S 41° 14′ 02′′ W). This URAD is supported by the Brazilian Government through the Ministry of Environment. The region experiences a dry climate, with temperatures varying from 21 °C (rainy season) to 39 °C (dry season) and an annual rainfall of around 690 mm. The main native plant species found in this region are ‘angico’ (*Anadenanthera colubrina*), ‘cumaru’ (*Amburana cearensis*), ‘faveleira’ (*Cnidoscolus phyllacanthus*), and ‘sabiá’ (*Mimosa caesalpiniaefolia*). The area of URAD represents a watershed that is susceptible to erosion if the soil surface is unprotected by vegetation. The soil type comprises ‘Litossolos’ arenic soils with slopes varying from 3 to 5%^41^, and the average erodibility is estimated at ~ 0.13 ha^−1^ h/ha MJ mm^42^. The land degradation in this region occurred through the exploration of native species (charcoal production) and the exposition of the soil surface to the direct impact of rain which promoted soil losses by erosion. To assess the effect of land degradation and potential restoration practices, we selected three main sites (~ 1 ha): (a) a degraded area; (b) a restored area; and a native area. The restoration practices started in 2019 applying the construction of successive stone dams (stone cords 30 cm high and wide) installed along the curves level and spaced 25 m between the lines that allowed the retention of rainwater, preventing soil erosion and increasing soil moisture. Additionally, the practices involved the restoration of riparian forests through the reintroduction of native species, coupled with the cultivation of annual local crops like maize and beans.

### Bulk soil sampling

Each main site was divided into four transects (~ 2500 m^2^) and three points per transect were used to collect soil samples (0–10 cm depth) and pooled together to obtain a composite sample per transect. The soil sampling was conducted in both dry (October 2022) and rainy (February 2023) seasons. Thus, a total of 24 soil samples were collected for DNA analysis. The sampling was done using sterile tools and in bags and kept at − 80 °C before DNA extraction. Soil properties (Table 1) were determined according to Embrapa^43^. Briefly, soil pH was determined in a 1:2.5 soil/water extract. Available P and exchangeable K were estimated by photometry and colorimetry, respectively, and SOM was determined by the wet combustion method. During each soil sampling, the soil temperature was measured for 5 min at 10 cm depth using a probe thermometer. The soil moisture content was estimated using the gravimetric method, being expressed as the mass of water per mass of dry soil.

**Table 1:** Main soil properties at different sites from URAD: native (Nat), degraded (Deg), and under restoration (Res).

Site	Temperature (°C)	Moisture (g g^−1^)	pH	K (mg kg^−1^)	P (mg kg^−1^)	SOM (g kg^−1^)	V (%)
Rainy							
Nat	28	0.18	4.9	92	10.9	16.8	71
Deg	26	0.08	5.1	53	5.1	3.49	62
Res	26	0.11	5.2	68	6.4	7.72	60
Dry							
Nat	33	0.002	4.8	98	11.6	15.7	69
Deg	40	0.001	5.1	59	5.8	3.14	63
Res	35	0.001	5.0	76	6.8	7.21	59

*SOM* soil organic matter, *V* basis saturation.

### DNA extraction and sequencing

As per the manufacturer’s guidelines, we employed a DNA extraction process from 0.5 g of soil utilizing the DNeasy PowerSoil Pro Kit by Qiagen, located in CA, USA, and subsequently stored it at − 20 °C. The DNA’s quality was assessed through spectrophotometry using the Nanodrop ND-1000. For the amplification of the V4 region of the 16S rRNA gene, we employed the 2X Kapa HiFi HotStart Ready Mix by Roche, headquartered in Pleasanton, CA, USA, along with the primer set 515F-Y (5′-GTGYCAGCMGCCGCGGTAA-3′) and 806R (5′-GGACTACHVHHHTWTCTAAT-3′)^44^. The amplification procedure included an initial step at 95 °C for 3 min, followed by 35 cycles at 98 °C for 20 s, 55 °C for 30 s, 72 °C for 30 s, and a final extension step at 72 °C for 5 min. Subsequently, we conducted a second index PCR employing the Nextera XT index kit v2 set by Illumina, based in San Diego, CA, USA. The resulting PCR products were purified using Agencourt AMPure XP beads, provided by Beckman Coulter, situated in Brea, CA, USA, and their quantification was performed using a Qubit fluorometer with the dsDNA BR Assay kit from Thermo Fisher Scientific, located in Waltham, Massachusetts, USA. The libraries were subjected to paired-end sequencing using the Illumina MiSeq Reagent Kit v2 (300-cycles, 2 × 150 bp) at the Genomic and Bioinformatic Facility Centre (CeGenBio) within the Federal University of Ceará, Brazil.

### Data analysis

 Raw reads were analyzed following the “Moving Pictures” tutorial” (v. 2023.7) from QIIME 2 (Quantitative Insight Into Microbial Ecology)^45^. Briefly, demultiplexed samples were imported and sequence quality control and feature table construction were done through DADA2 pipeline. We used a Q-score = 30 for quality filter (Table S1). To subsequently analyze, we adopted a sampling depth of 9537. Afterward, QIIME 2 artifacts were loaded into phyloseq package in RStudio and created alpha (observed ASVs, and Shannon) and beta (Non-metric Multi-Dimensional Scaling plot, NMDS) diversity metrics. PERMANOVA was used to test special differences between bacterial communities across treatments (degraded, native, and restoration) and seasons (dry and rainy), while Kruskal–Wallis, a non-parametric test, was adopted to test differences between samples (averages). Microbial composition was obtained against SILVA v. 138 database and relative abundance (%) was generated in phyloseq through histogram plots of phyla and family levels. Co-occurrence network analyses were conducted to evaluate the interrelationships within bacterial communities across three distinct URAD treatments. These analyses involved examining positive and negative correlations, as well as assessing the number of nodes and edges in the networks. Non-random co-occurrence analyses were specifically conducted using SparCC. To perform these co-occurrence analyses, the Python module ‘SparCC’ was utilized, and subsequent network visualization and statistical measurements were computed through Gephi. The assessment of niche occupancy, specifically the distribution of generalists and specialists within each URAD treatment, was determined using the multinomial species classification method. This analysis was conducted in R, utilizing the ‘vegan’ package and the ‘clamtest’ function, with a significance level set at 5%. FASTQ raw data files have been deposited to the NCBI database under BioProject number SUB14173999.

### Supplementary Information

Supplementary Table S1.

## Data Availability

The datasets used and/or analysed during the current study available from the corresponding author on reasonable request.
