# Supplementary material for: Short-term restoration practices change the bacterial community in degraded soil from the Brazilian semiarid
Source: Sci Rep. 2024 Mar 21;14:6845. doi: 10.1038/s41598-024-57690-y (PMC10957980; doi:10.1038/s41598-024-57690-y)

**Short-term restoration practices change the bacterial community in degraded soil from the Brazilian semiarid**

Davila Esmelinda Oliveira Silva, Romario Martins Costa, Janaira Rocha Campos, Sandra Mara Barbosa Rocha, Arthur Prudencio de Araujo Pereira, Vania Maria Maciel Melo, Francisca Andrea Silva Oliveira, Francisco de Alcantara Neto, Lucas William Mendes, Ademir Sergio Ferreira Araujo

**Supplementary Table S1.** Filtering and read numbers


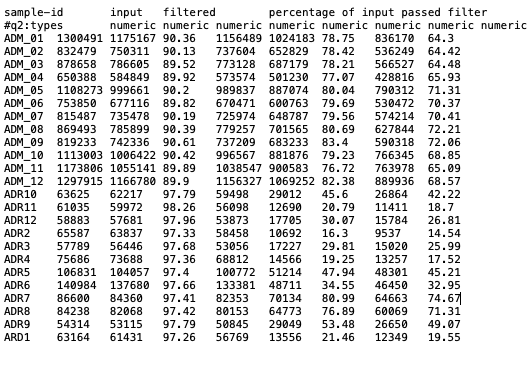

Supplement: Supplementary file 1 — Supplementary Table S1. [file 41598_2024_57690_MOESM1_ESM.docx]
